# Lung Cancer in Railroad Workers Exposed to Diesel Exhaust

**DOI:** 10.1289/ehp.7195

**Published:** 2004-08-05

**Authors:** Eric Garshick, Francine Laden, Jaime E. Hart, Bernard Rosner, Thomas J. Smith, Douglas W. Dockery, Frank E. Speizer

**Affiliations:** ^1^Pulmonary and Critical Care Medicine Section, Medical Service, Veterans Affairs Boston Healthcare System, Boston, Massachusetts, USA; ^2^Channing Laboratory, Department of Medicine, Brigham and Women’s Hospital, Harvard Medical School, Boston, Massachusetts, USA; ^3^Exposure, Epidemiology and Risk Program, Department of Environmental Health, Harvard School of Public Health, Boston, Massachusetts, USA

**Keywords:** diesel exhaust, lung cancer, occupational exposure

## Abstract

Diesel exhaust has been suspected to be a lung carcinogen. The assessment of this lung cancer risk has been limited by lack of studies of exposed workers followed for many years. In this study, we assessed lung cancer mortality in 54,973 U.S. railroad workers between 1959 and 1996 (38 years). By 1959, the U.S. railroad industry had largely converted from coal-fired to diesel-powered locomotives. We obtained work histories from the U.S. Railroad Retirement Board, and ascertained mortality using Railroad Retirement Board, Social Security, and Health Care Financing Administration records. Cause of death was obtained from the National Death Index and death certificates. There were 43,593 total deaths including 4,351 lung cancer deaths. Adjusting for a healthy worker survivor effect and age, railroad workers in jobs associated with operating trains had a relative risk of lung cancer mortality of 1.40 (95% confidence interval, 1.30–1.51). Lung cancer mortality did not increase with increasing years of work in these jobs. Lung cancer mortality was elevated in jobs associated with work on trains powered by diesel locomotives. Although a contribution from exposure to coal combustion products before 1959 cannot be excluded, these results suggest that exposure to diesel exhaust contributed to lung cancer mortality in this cohort.

Since the 1970s, there has been concern that inhalation of diesel exhaust may cause lung cancer in humans. Diesel exhaust particles are respirable and contain mutagenic and carcinogenic compounds on a carbonaceous core (Schenker 1980). In > 35 studies of workers exposed to freshly generated diesel exhaust, an excess lung cancer risk in the range of 20–50% has been observed (Bhatia et al. 1998; Lipsett and Campleman 1999). However, the nature of the risk between exposure to diesel exhaust and human lung cancer is still being debated (Diesel Epidemiology Working Group 2002). Only limited information is available linking job title to duration and intensity of exposure (Garshick et al. 1987a, 1988; Steenland et al. 1990; Zaebst et al. 1991). Few studies have had occupational histories and follow-up over enough years to assess risk because lung cancer may develop only after many years of exposure and years of latency.

Exposure to high levels of diesel particles has produced lung cancer in rats but has not consistently produced lung cancer in other rodent species (Heinrich et al. 1986; Mauderly et al. 1987). However, lung cancer also has been produced in rats exposed to high levels of fine particles devoid of organics (Borm et al. 2004; Nikula et al. 1995). In these studies, particle clearance mechanisms became overloaded, and pulmonary inflammatory changes were noted. These responses have not been associated with lung cancer in humans. Therefore, the U.S. Environmental Protection Agency (EPA) has described the risk of diesel-exhaust–related lung cancer based on human epidemiologic studies (U.S. EPA 2002).

After World War II, there was a rapid transition by U.S. railroads from steam to diesel locomotives. In 1946, 10% of the locomotives in service were diesel powered, but by 1959 the proportion had increased to 95% diesel (U.S. Department of Labor, Bureau of Labor Statistics 1972). We previously published a retrospective cohort mortality study of lung cancer among 55,407 railroad workers with 10–20 years of work experience in 1959 who had mortality follow-up through 1980. Occupational exposures to diesel were categorized based on an industrial hygiene survey (Woskie et al. 1988a, 1988b). Workers 40–44 years of age, with the greatest likelihood of diesel exposure, had a relative risk (RR) of lung cancer mortality of 1.45 [95% confidence interval (CI), 1.11–1.89] (Garshick et al. 1988). Older workers had a lower lung cancer risk. It was later recognized that the mortality ascertainment was incomplete between 1977 and 1980. Similar results were obtained limiting follow-up through 1976 (Crump 1999; Larkin et al. 2000). Follow-up of this cohort has continued, and in this article we present an assessment of lung cancer mortality risk over a 38-year period (through 1996) with improved mortality information and additional work history data.

## Materials and Methods

### Population description.

The U.S. Railroad Retirement Board (RRB) has maintained a computerized record of work history since 1959. In 1981, men 40–64 years of age with 10–20 years of railroad service in 1959 were selected for data extraction. Based on job in 1959, we identified 39 job codes with exposure to diesel exhaust characterized during an industrial hygiene survey (Woskie et al. 1988a, 1988b). We sampled 56,208 workers in these job codes, including *a*) every third engineer (engineers and firemen), *b*) every third conductor (conductors, brakemen, and hostlers), *c*) all shop workers (shop supervisors, machinists, and electricians), and *d* ) a referent group of less-exposed workers (ticket agents, station agents, and signal maintainers, and every fourth clerk). By design, approximately 75% of the workers in the sample were in diesel-exposed jobs and 25% were in low- or no-exposure jobs. The RRB provided a listing of yearly job code, months of railroad service, and mortality information through 1980. The final analytic cohort included 55,407 white males. We later determined that the job codes of shop workers were not specific to work in areas with locomotive exhaust, so diesel exposures of these job codes could not be specified. In addition, it was possible that some of these workers had previous asbestos exposure in steam engine repair shops. Therefore, we considered shop workers separately from the “exposed group” of conductors and engineers. Other workers with potential asbestos exposure were the hostlers (*n* = 779 in 1959). These workers may have had exposure while driving locomotives in and out of repair facilities during the steam locomotive era. Analyses were conducted with and without these workers based on job in 1959.

### Work history and mortality update through 1996.

The Brigham and Women’s Hospital and Veterans Affairs Boston Healthcare System institutional review boards approved the protocol. The RRB provided updated work history information through 1996 for workers meeting the original extraction criteria, and linkage with the original database was possible for 55,016 subjects (99.2%). Updated mortality information was determined from RRB records, the Social Security Death Master (SSDM), and Health Care Financing Administration records. We excluded 43 subjects with < 10 years of railroad service or found to have no job reported on enrollment in 1959, leaving 54,973 subjects.

### Cause of death determination.

For subjects known to have died, cause of death [*International Classification of Diseases*, *9th Revision* (ICD-9); World Health Organization (WHO) 1978] was obtained from a search of the National Death Index (NDI) between 1979 (the first year NDI was available) and 1996. For subjects without a valid match and for all others without specific cause of death, efforts were made to obtain a death certificate. State of death was obtained from the SSDM and from a manual search of RRB records. Underlying cause of death was coded using computer packages from the National Center for Health Statistics (1997a, 1997b, 1997c). A nosologist blinded to exposure status coded death certificates not accepted by the program. ICD-8 (*8th Revision*; WHO 1967) cause of death from the original 1959–1980 database was converted to ICD-9 codes based on three-digit category. To include all possible cases, lung cancer mortality (ICD-9 code 162) was defined by the underlying cause of death or by lung cancer appearing elsewhere on the death certificate or NDI record. This is appropriate because lung cancer is usually rapidly fatal after diagnosis.

### Statistical analysis.

To evaluate completeness of mortality ascertainment, standardized mortality ratios (SMRs) were calculated as the ratio of the observed deaths compared with the number of deaths expected based on calendar year and 5-year age-specific mortality rates for U.S. white men. Proportional hazard analyses (SAS, version 8; SAS Institute, Inc., Cary, NC) assessed lung cancer mortality with calendar month as the time axis. Person-time was calculated from 1 January 1959 to date of death or to 31 December 1996, whichever was earlier. Hazard ratios (referred to as RRs) and 95% CIs are presented separately for engineers, conductors, and shop worker groups and for the engineer and conductor groups combined compared with unexposed workers based on job code on entry into the study in 1959. Because years of work in a diesel-exposed job were related to age in 1959 (Table 1), we assessed effect modification using interaction terms with 5-year age groups and exposure-job group in 1959, as in our previous study (Garshick et al. 1988). To account for a healthy worker survivor effect, we included time-varying variables for total years worked and for years off work (usually time after retirement) in survival models. Age was controlled by stratification in 1-year categories. First year of work was available for workers starting in 1947 or later and was estimated based on total months of service for those employed before 1947.

The association of lung cancer mortality with duration of exposure as a surrogate for cumulative dose was assessed as a time-varying covariate, based on yearly job code and service months, starting in 1959 in the combined engineer and conductor groups, and was grouped in 5-year exposure categories. The classification of exposure after 1959 in jobs for which no industrial hygiene sampling was done (4.6% of yearly job codes) was based on similarities in work locations and duties to the original 39 job codes. Models with 5-, 10-, and 15-year exposure lags were assessed, meaning that exposure in the year of death and in the previous 4, 9, and 14 years, respectively, was not included in the calculation of exposure duration. To assess appropriateness of each exposure lag, we also assessed the association of lung cancer mortality with exposure 5, 10, and 15 years before death. An indicator variable was included to account for work in a shop job code between 1959 and 1996.

## Results

### Cohort mortality.

There were 43,593 deaths between 1959 and 1996 in the 1,364,382 person-years of follow-up. There were 21,639 deaths in the original analysis period (1959–1980) and 21,954 deaths in the additional follow-up period (1981–1996). Of 2,302 deaths from 1959 to 1980 not identified in the original analysis, 98% occurred between 1977 and 1980, the time period with known incomplete mortality ascertainment. Cause of death was defined for 21,116 (98%) deaths from 1959 to 1980 and for 21,670 (99%) deaths from 1981 to 1996. The major causes were circulatory system diseases (ICD-9 codes 390–459, *n* = 21,779, 50%), malignant neoplasms (ICD-9 codes 140–208, *n* = 10,558, 24%), and respiratory system diseases (ICD-9 codes 460–519, *n* = 3,878, 9%). Lung cancer was identified as the underlying cause in 4,021 deaths and as a contributing cause in 330 deaths (4,351 total lung cancer deaths).

The SMR in 1959 was 0.81, consistent with a healthy worker effect (Choi 1992; Li and Sung 1999). Over time, the SMR increased such that by 1967, the yearly SMR had risen to 1.01. Overall, the SMR for all deaths was 1.01, indicating that death ascertainment was effectively complete.

### Job histories.

The distribution of lung cancer deaths and years worked by age and job group is presented in Table 1. Career paths within the railroad industry are stable. Over the entire 38 years of follow-up, only 126 workers transferred into engineer or conductor job codes and 284 workers transferred into shop worker job codes. Of unexposed workers in 1959, 97% remained unexposed through the follow-up period. As expected, younger workers at study entry had greater potential for longer years of work (i.e., exposure) compared with older workers.

### Total mortality.

Total mortality was elevated for exposed workers, defined as working as either engineers or conductors in 1959, compared with unexposed workers (clerks and signal maintainers; RR = 1.17; 95% CI, 1.14–1.20), adjusting for total years worked, time since last worked, and attained age. The RR of mortality due to circulatory system diseases was 1.13 (95% CI, 1.09–1.16); that due to all respiratory system diseases, including chronic obstructive pulmonary disease (COPD) and allied conditions (ICD-9 490–496), was 1.31 (95% CI, 1.21–1.42); and that due to COPD and allied conditions alone was 1.41 (95% CI, 1.27–1.55). The RR due to lung cancer was 1.40 (95% CI, 1.30–1.51). We explore the excess risk due to lung cancer in further detail below.

### Lung cancer mortality.

There was some evidence of effect modification of the lung cancer–diesel exposure relationship by age at entry to the study. Defining exposure by job title in 1959, independently engineers and conductors 40–44, 45–49, 50–54, and 55–59 years of age at study entry had elevated lung cancer risks (Table 2). Among workers 60–64 years of age at entry, the risk was elevated only in the conductor group but not statistically significantly. When the exposed groups were combined, the risk was greatest among the youngest workers (40–44 years of age at study entry) but still significantly elevated for all exposed workers < 60 years of age at study entry. The relative risk for shop workers was elevated only in the 55–59 year age group.

Lung cancer mortality was inversely related to total years worked (RR = 0.97; 95% CI, 0.96–0.98 per year). Relative risk of dying was greatest within the first year after leaving work (RR = 6.14; 95% CI, 5.27–7.14) and decreased with subsequent years, ranging from 2.98 (95% CI, 2.57–3.45) for 2–5 years, 2.74 (95% CI, 2.31–3.26) for 6–10 years, 2.54 (95% CI, 2.05–3.14) for 11–15 years, 2.39 (95% CI, 1.85–3.10) for 16–20 years, and 2.34 (95% CI, 1.72–3.31) for ≥21 years off work. There was no significant effect modification of years on or off work based on diesel exposure (data not shown).

We assessed the relationship between cumulative years of work as a surrogate for diesel exhaust exposure and lung cancer risk, controlling for attained age, any shop work, total years worked, and time since last worked in models without an exposure lag, and with lags of 5, 10, and 15 years (Table 3). Lung cancer mortality was significantly associated with a diesel exhaust exposed job group regardless of the exposure lag model, but risk did not increase with years of exposure. Restriction of the cohort to subjects who survived beyond the last year worked and stratifying on retirement time gave similar results (results not shown).

To assess the significance of exposure in the years before death on lung cancer risk, we included an indicator variable for work in the 5, 10, and 15 years before death in the exposure lag models in Table 3. In the 5 years before death, lung cancer mortality was not significantly elevated compared with unexposed workers (RR = 1.14; 95% CI, 0.85–1.54). RR was 1.26 (95% CI, 1.06–1.50) for exposure within 10 years before death and 1.40 (95% CI, 1.23–1.59) for exposure within 15 years before death. These results suggest that it is appropriate to exclude exposure in the 5 years before death in the assessment of lung cancer mortality. Assuming a 5-year exposure lag before death, lung cancer mortality associated with any exposure after 1959 was 1.40 (95% CI, 1.30–1.51), the same result obtained based on work in an exposed job based on job at entry.

We considered the possibility that lung cancer mortality risk varied based on selection of the reference group. Signal maintainers (*n* = 3,536 based on job at entry; 259 lung cancer deaths) who worked on the track were considered separately from ticket agents, station agents, and clerks, who worked in offices (*n* = 10,411 based on job at entry; 704 lung cancer deaths), thus defining a non-office-based, blue-collar control group. Regardless of which comparison group was used, similar lung cancer mortality risks were observed. Excluding the hostlers, potentially exposed to asbestos, from analysis did not change the results.

## Discussion

We present a retrospective assessment of lung cancer mortality for 38 years of follow-up in a large cohort of railroad workers, finding elevated risk among engineers, firemen, conductors, and brakemen, job categories identified as diesel exposed. Disregarding exposure in the 5 years before death, the RR for these workers compared with workers without regular work in an exposed job was 1.40 (95% CI, 1.30–1.51). Unlike the original findings of greatest risk in younger workers, lung cancer mortality was elevated to a similar extent regardless of age at entry (in 1959) except in workers 60–64 years of age. Thus, excess risk was not limited to workers with the greatest opportunity for exposure because of their being younger at the start of the diesel era. Finally, there was no evidence of an increased risk with increasing years of work (the exposure surrogate) in a job with exposure to diesel exhaust.

Our observation of lung cancer risk is similar to the risk noted by others in the literature. In > 35 studies of workers with occupational exposure to diesel exhaust, excess risk of lung cancer is consistently elevated by 20–50% (reviewed in Bhatia et al. 1998; Lipsett and Campleman 1999). Most occupational studies rely on a single report of job title to define exposure. In this study, job title was available for each year of follow-up, and jobs with exposure to diesel emissions were defined by an industrial hygiene survey. These results are similar to smoking-adjusted RRs attributable to fine particulate air pollution on lung cancer in prospective population-based cohorts (Dockery et al. 1993; Pope et al. 2002) and risk of lung cancer attributable to vehicle exhausts in urban settings (Nyberg et al. 2000). Effects for cardiovascular and respiratory disease mortality are also consistent with observations reported by population-based studies (Dockery et al. 1993; Pope et al. 2002).

Although we originally reported that lung cancer risk increased with increasing years of work in diesel-exposed jobs (Garshick et al. 1988), subsequent reanalyses of these data, with adjustment for attained age, indicated decreased risk with more years worked (Crump 1999; Health Effects Institute 1999). This inverse association with exposure duration could be explained by a healthy worker survivor effect. Analysis in this updated cohort with longer follow-up also indicates that lung cancer mortality is inversely related to total years worked. The possibility that the healthy worker survivor effect influences the assessment of mortality had not been considered previously. Although methods for controlling for the healthy worker survivor effect have been proposed, it is uncertain whether full adjustment by statistical methods is possible. It was not possible to implement methods suggested by Robins (1987) because there was little change in exposure status, and retirement patterns were stable. Other methods to adjust for healthy worker effects (Arrighi and Hertz-Picciotto 1993, 1994) consider employment status and exposure lag models to exclude recent exposure. With overall employment duration and employment status considered, the relationship between lung cancer risk and years of work in a diesel-exposed job was elevated regardless of exposure duration (Table 3). Restriction of the cohort to subjects who survived beyond the last year worked and stratification on retirement time also gave similar results. We also conducted alternative survival analyses (compared with proportional hazards methodology) employing recently developed techniques in which time to an event is modeled using “first hitting time” methodology (Lee and Whitmore 2003; Lee et al. 2004). Using these methods, there was evidence of a healthy worker survivor effect, with an elevated risk of lung cancer mortality among train crews (Lee et al. 2004).

Exposures before 1959 and changes in exposure patterns could also modify a relationship between years of work and lung cancer mortality. An expectation of increasing risk with years of exposure implicitly assumes that the exposure intensity is approximately constant across years. Diesel locomotive emissions changed throughout the follow-up period. Explicit exposure data are not available, but the first diesel engines (1940s through 1950s) were said to be “smokier” than later locomotives (Woskie et al. 1988b). Cleaner locomotives were introduced in the early 1960s and the 1980s. Although diesel engines are known to produce mainly fine and ultrafine particles, similar information is not available on coal-fired locomotives. Temporal changes in diesel and other combustion-related emissions might contribute to the lack of an exposure–response relationship based on duration of exposure in the train crews.

Because all workers were employed in 1959 and had exposures in the previous 10–20 years, we could not assess whether work exclusively during the diesel or steam locomotive era or with early diesel locomotives differentially influenced mortality. However, in a case–control study using RRB records to determine deaths in 1981–1982, workers > 65 years at death were exposed mainly to steam engine emissions, and younger workers mainly to diesel engine emissions. In the older group, work in diesel-exposed jobs was not associated with lung cancer mortality, whereas the RR was significantly elevated for the younger group. In the present study, the oldest workers (60–64 years of age at study entry) had the fewest years of work after 1959 and the lowest mortality due to lung cancer. These results suggest that introduction of diesel locomotives significantly contributed to lung cancer mortality in the cohort.

Small RRs may be affected by uncontrolled confounding, such as differences in cigarette smoking habits in subjects with and without diesel exposure. In this retrospective cohort, individual data on smoking history are not available. To minimize the possible effect of uncontrolled confounding by smoking, efforts were made to include only workers of similar socioeconomic class, a known correlate of smoking habits (Brackbill et al. 1988; Stellman et al. 1988). Further, estimates were similar when the reference group was restricted to signal maintainers, potentially a more blue-collar unexposed group. Smoking rates vary by birth cohort (Burns et al. 1997). However, all analyses are stratified by age; thus, birth cohort is controlled for.

In our previous case–control study using RRB records (Garshick et al. 1987a), smoking history was obtained from next of kin, and crude and smoking-adjusted effects of exposure were similar. With the distribution of job-specific smoking habits from the case–control study and a survey of 514 white male workers employed by a small railroad in 1982 (Garshick et al. 1987b), we calculated age- and job-specific smoking adjustment factors using Schlesselman and Axelson methods (Axelson and Steenland 1988; Larkin et al. 2000; Schlesselman 1978). These factors, the ratio (diesel exposed:unexposed) of literature-based lung cancer risks weighted by job-specific smoking behavior generally ranged from 1.1 to 1.2 (Larkin et al. 2000). Other investigators have reported similar factors (Blair et al. 1985; Levin et al. 1990; Siemiatycki et al. 1988). Dividing the observed RR for lung cancer for the present study by these factors attenuated the RR to between 1.17 and 1.27. These estimates are consistent with other literature-based smoking-adjusted risks attributable to diesel exhaust, traffic emissions, and air pollution (Dockery et al. 1993; Nyberg et al. 2000; Pope et al. 2002; Steenland et al. 1990). This indirect method is limited in adjusting for smoking by assuming no interaction between diesel exposure and smoking, but there are insufficient data to assess this possibility.

Respiratory disease mortality, including from COPD and allied conditions, was also associated with exposure. The predominant cause of these diseases is cigarette smoking, possibly providing evidence of confounding by smoking in our lung cancer analyses. However, smoking-adjusted cohort studies show that occupational exposures to dusts and fumes are also associated with chronic respiratory symptoms and airflow obstruction (Garshick et al. 1996, 2003; Hnizdo et al. 2002). Studies of workers specifically exposed to diesel exhaust indicate that there is an increase in respiratory symptoms and a reduction in pulmonary function with exposures (U.S. EPA 2002). Controlled studies of human exposures to diesel exhaust and to other fine particles results in pulmonary inflammatory changes (Ghio et al. 2000; Salvi et al. 1999). Therefore, exposure to diesel exhaust from operating trains may in fact lead to an increased risk of chronic respiratory disease mortality, independent of smoking.

Factors other than smoking that might modify the risk of lung cancer seem unlikely to contribute further uncertainty to these results (Alavanja et al. 2001; Henley et al. 2002; Olson et al. 2002). These factors are much less significant than smoking and not expected to be related to exposure. Controlling for the healthy survivor effect by considering the ability to work (years of work) and live into retirement (time off work) in the regression models also may reduce uncontrolled confounding by other lifestyle-related factors and might further address adjustment for smoking behavior. Death certificates were used to identify causes of death. Death certificates may overascertain rather than underascertain primary lung cancer (Bauer and Robbins 1972; Goldman et al. 1983; Jimenez et al. 1975; Kircher et al. 1985; Percy et al. 1981; Rosenblatt et al. 1971). This type of misclassification is likely to be random with respect to exposure and would make the effect of exposure harder to detect.

## Conclusion

Lung cancer mortality in workers in diesel-exposed jobs was elevated in this cohort. It is unlikely that this association is explained by uncontrolled confounding. We believe there was no relationship between years of exposure and lung cancer risk because of the healthy worker survivor effect, the lack of information on historical changes in exposure, and the potential contribution of coal combustion products before the transition to diesel. However, these results indicate that the association between diesel exhaust exposure and lung cancer is real. These results along with previous studies of lung cancer and diesel exhaust support current efforts to reduce emissions in both occupational and general environmental settings. Studies designed to provide quantitative exposure estimates are needed to better quantify health risks, including those related to more contemporary diesel engines used in light- and heavy-duty diesel on-road vehicles.

## Figures and Tables

**Table 1:** Distribution of the cohort by exposure categories, duration of service, and exposure presented by age (years) at baseline (1959).

Characteristic	40–44 (*n* = 19,794)	45–49 (*n* = 13,874)	50–54 (*n* = 9,820)	55–59 (*n* = 7,216)	60–64 (*n* = 4,269)	Total (*n* = 54,973)
1959 job groups						
Unexposed	4,911	3,132	2,601	1,989	1,314	13,947
Engineers*^a^*	4,002	2,834	1,855	1,394	836	10,921
Conductors*^b^*	7,204	5,074	2,898	1,916	971	18,063
Shop*^c^*	3,677	2,834	2,466	1,917	1,148	12,042
Lung cancer deaths						
Unexposed	292	241	199	138	93	963
Engineers	328	257	189	113	41	928
Conductors	556	475	267	173	80	1,551
Shop	241	220	182	184	82	909
Total	1,417	1,193	837	608	296	4,351
Years of exposure						
1959 to retirement*^d^*						
0 (unexposed)	4,747	3,016	2,530	1,942	1,302	13,537
1–< 5	984	831	689	842	1,115	4,461
5–< 10	1,082	1,005	946	1,694	647	5,374
10–< 15	1,450	1,727	2,670	791	47	6,685
15–< 20	4,519	4,117	451	6	0	9,093
≥20	3,219	267	11	0	0	3,497
Any shop	3,793	2,911	2,523	1,941	1,158	12,326
Duration of employment						
Retirement year						
Median	1977	1974	1971	1966	1962	
IQR	1972–1979	1970–1975	1967–1972	1964–1969	1961–1965	
Hire date > 1945 (%)	28.9	22.1	20.5	17.2	13.5	
Years of service (mean ± SD)	32.9 ± 6.4	30.5 ± 5.7	27.5 ± 7.8	24.7 ± 4.0	22.0 ± 3.4	

IQR, interquartile range.

a Engineers, firemen.

b Conductors, brakemen, hostlers.

c Shop workers.

d Years of work in engineer or conductor group; “any shop” refers to any worker in a shop-worker job between 1959 and retirement.

**Table 2:** Interaction of 5-year age group (years) and job title at study entry in 1959 and RRs of lung cancer mortality 1959–1996 for engineers, conductors, and shop workers compared to unexposed workers.

	40–44	45–49	50–54	55–59	60–64
Unexposed					
Cases	292	241	199	138	93
Person-years	148,701	83,949	58,483	36,992	20,072
RR	Reference	Reference	Reference	Reference	Reference
Engineer					
Cases	328	257	189	113	41
Person-years	116,129	72,820	40,190	25,261	13,041
RR (95% CI)	1.59 (1.35–1.86)	1.36 (1.14–1.63)	1.51 (1.24–1.84)	1.27 (0.99–1.63)	0.71 (0.49–1.02)
Conductor					
Cases	556	475	267	173	80
Person-years	209,897	130,084	63,127	34,421	14,219
RR (95% CI)	1.43 (1.24–1.65)	1.37 (1.17–1.60)	1.32 (1.10–1.58)	1.38 (1.11–1.73)	1.24 (0.92–1.67)
Shop worker					
Cases	241	220	182	184	82
Person-years	109,670	75,286	56,187	36,902	18,952
RR (95% CI)	1.10 (0.93–1.31)	0.99 (0.82–1.18)	0.92 (0.75–1.13)	1.31 (1.05–1.63)	0.88 (0.65–1.18)
Engineers and conductor groups combined					
Cases	884	732	456	286	121
Person-years	326,026	202,903	103,317	59,682	27,260
RR (95% CI)	1.49 (1.30–1.70)	1.37 (1.18–1.58)	1.39 (1.18–1.64)	1.34 (1.09–1.64)	0.99 (0.75–1.30)

Models are adjusted for age, years of employment, and time off work as time-dependent covariates.

**Table 3:** RR of lung cancer mortality based on cumulative years of work in an engineer or conductor job group, adjusting for age and work in any shop category.

Exposure lag	Unexposed	0 to < 5 years	5 to < 10 years	10 to < 15 years	15 to < 20 years	≥20 years
None						
Cases	922	334	468	665	782	241
Person-years	338,088	171,943	172,565	158,565	166,085	52,493
RR (95% CI)	Reference	1.35 (1.17–1.54)	1.43 (1.26–1.60)	1.58 (1.42–1.75)	1.30 (1.17–1.44)	1.24 (1.05–1.45)
5 years						
Cases	1,008	391	484	618	707	204
Person-years	480,468	150,143	145,607	126,189	121,884	35,448
RR (95% CI)	Reference	1.41 (1.24–1.61)	1.39 (1.23–1.56)	1.51 (1.35–1.68)	1.33 (1.19–1.49)	1.31 (1.10–1.56)
10 years						
Cases	1,211	449	479	587	544	142
Person-years	613,828	127,484	119,723	96,675	82,041	19,986
RR (95% CI)	Reference	1.49 (1.31–1.69)	1.27 (1.12–1.44)	1.50 (1.33–1.68)	1.29 (1.14–1.46)	1.50 (1.22–1.85)
15 years						
Cases	1,532	456	511	499	360	54
Person-years	733,767	105,240	95,345	70,145	48,102	7,141
RR (95% CI)	Reference	1.31 (1.15–1.49)	1.30 (1.15–1.47)	1.38 (1.22–1.57)	1.34 (1.15–1.58)	1.40 (1.02–1.92)

Models are adjusted for age, years of employment, and time off work as time-dependent covariates. In the exposure lag models, work in an engineer or conductor job group 5, 10, and 15 years before death is not included as exposure.
